# From Archipelago to Pandemic Battleground: Unveiling Indonesia’s COVID-19 Crisis

**DOI:** 10.1007/s44197-023-00148-7

**Published:** 2023-09-14

**Authors:** Biyan Nathanael Harapan, Triswan Harapan, Lenny Theodora, Nadia Ayurini Anantama

**Affiliations:** 1 Ludwig-Maximilians-Universität München, Munich, Germany; 2 Complementary Cardiovascular Clinic (CCV Clinic), Tangerang Selatan, Indonesia; 3 Tangerang Selatan, Indonesia; 4 Leipzig University, Leipzig, Germany

**Keywords:** COVID-19, Coronavirus, SARS-CoV-2, Epidemiology, Indonesia, Vaccination

## Abstract

The coronavirus disease 2019 (COVID-19) pandemic has posed unprecedented challenges to countries worldwide, including Indonesia. With its unique archipelagic geography consisting of more than 17,000 thousand islands, Indonesia faces unique complexities in managing the spread of the virus. Based on existing literature, this review article elaborates on key issues that have shaped Indonesia’s COVID-19 response. The article begins by examining the early stages of the COVID-19 pandemic in Indonesia, along with the implementation of various preventive measures and the impact of the virus on public health. This article examines how Indonesia’s socio-economic factors have generally influenced its healthcare system and further delves into the COVID-19 response strategies implemented by the Indonesian government and public health authorities as well as overall crisis preparedness. It discusses the actions taken to control the spread of the virus, including testing strategies and vaccination efforts. The difficulties encountered in implementing these measures are presented. In conclusion, this review article provides a comprehensive understanding of the COVID-19 crisis in Indonesia, covering facts on multiple dimensions ranging from the timeline of the pandemic to vaccination efforts, epidemiology, socio-economic implications, testing strategies, mobility patterns, public holidays, the impact of working from home, and the utilization of complementary and alternative medicine in addition to the standard of care for COVID-19. The insights gained from this article can complement future strategies for pandemic management and response in Indonesia and other countries facing similar challenges.

## Introduction

The coronavirus disease 2019 (COVID-19) pandemic has emerged as one of the most wide-ranging challenges in recent years, impacting nations across the globe. Among the countries grappling with the multifaceted implications of this global health crisis is Indonesia, the world’s largest archipelagic state with a population exceeding 270 million and, therefore, the fourth most populous country in the world [1]. As the virus continues to evolve and disrupt lives, it is essential to examine and reflect upon the COVID-19 situation in Indonesia, exploring the unique dynamics, problems, and responses that have shaped the country's journey.

Indonesia has faced numerous hurdles in controlling the spread of the virus due to its geographical [2] and cultural context. 6000 inhabited islands and diverse populations have posed difficulties in implementing uniform health measures and ensuring equal access to healthcare services across the entire nation. In addition, the reliance on air and sea transportation for inter-island travel has further complicated containment efforts. The difficulties faced by Indonesia’s healthcare system, such as strained resources, limited healthcare infrastructure, and healthcare disparities across different regions are also important factors [3]. In this review article, we delve into the COVID-19 situation in Indonesia, aiming to provide insights and observations on the key dimensions that have shaped the country’s COVID-19 response.

## Background and Key Issues of the COVID-19 Pandemic in Indonesia

### Epidemiology and Timeline

On January 30, 2020, the World Health Organization (WHO) declared the outbreak of the novel coronavirus, severe acute respiratory syndrome coronavirus 2 (SARS-CoV-2), as a Public Health Emergency of International Concern (PHEIC) [4]. On March 2, 2020, Indonesia confirmed its first case of COVID-19, marking the beginning of the pandemic in the country [5]. The virus quickly spread throughout the nation, leading to a surge in infections and a remarkable increase in the number of COVID-19 cases while simultaneously placing a strain on the healthcare system. The Indonesian government promptly responded by implementing several measures, such as travel restrictions, the closure of public spaces to mitigate the spread of the virus, testing, contact tracing and social distancing protocols [6]. Despite these efforts, challenges arose due to delays in contact tracing, public compliance issues among the population and limited testing capacity [7]. Consequently, the initial response to the pandemic in the first few months in Indonesia highlighted the need for, among others, enhanced public awareness to effectively control the virus’s transmission and increased testing capacity.

The COVID-19 pandemic has had a significant impact on the epidemiological landscape in Indonesia. As of August 9, 2023, the total number of confirmed cases in Indonesia has surpassed 6.8 million, with over 161,900 deaths noted, indicating widespread transmission across the country [8]. The number of unconfirmed cases and deaths is suspected to be significantly higher [9].

During the first wave of the COVID-19 pandemic in Indonesia, the country faced significant challenges in containing the virus. Following the confirmation of the first cases in early March [10], there was a rapid increase in the number of infections throughout the country. One month later (April 2, 2020), Indonesia has reached 1790 confirmed cases with 170 deaths [11]. As of May 22, 2020, the Indonesian government provided data indicating that there was a total of 21,430 confirmed COVID-19 cases. Among these cases, 14,413 individuals (69.3%) were receiving medical treatment, while 5057 individuals (24.32%) had successfully recovered. Tragically, the number of fatalities amounted to 1326 individuals (6.4%) [12]. By September 21, 2020, 248,852 COVID-19 cases have been detected [13]. The epicenter of the outbreak was in Jakarta, the capital city of Indonesia whose metropolitan area is resident to 31.24 million people according to the Indonesian 2020 Census [14, 15], where a high population density and frequent international travel contributed to the virus’s rapid spread [16]. The virus continued to pose relevant health, social, and economic challenges, necessitating sustained efforts in surveillance, vaccination, testing, and treatment.

The examination of genomic data demonstrated that the first wave in Indonesia (up to May 15, 2021) was predominantly characterized by the presence of the B.1.466.2 lineage, while the second wave (around July 2021) was dominated by the prevalence of Delta variants, during which the number of confirmed cases surpassed those in the first wave of COVID-19 pandemic [17]. As the Delta variant spread, Indonesia became Asia’s new pandemic epicenter [18].

The further course of the pandemic was primarily defined by efforts to contain the viral spread via testing and vaccinations (as described below). On May 5, 2023, a significant milestone was reached in the global fight against the COVID-19 pandemic as the WHO made the decision to officially end the declaration of COVID-19 as a PHEIC [19]. While the termination of the PHEIC declaration is an encouraging step forward, it is important to acknowledge that the fight against COVID-19 is far from over. For almost a year now, many countries have started easing their public health guidelines, specifically in relation to wearing face masks during outdoor activities. The rationale behind this decision is the overall decline in reported cases of COVID-19. However, the emergence of new Omicron sub-variants, specifically BA.4 and BA.5, has been observed [20]. On June 21, 2023 the Indonesian president Joko Widodo announced the end of the COVID-19 pandemic status [21]. As Indonesia transitions into the endemic period, policies were released to uphold health protocols and the government continues to urge the public to remain vigilant and maintain hygiene and health habits as taught during the COVID-19 pandemic [22].

To understand the evolving epidemiology of COVID-19 in Indonesia, ongoing monitoring and surveillance are essential aspects that have to be considered. By closely identifying hotspots, tracking the number of cases, and analyzing demographic trends, public health authorities can tailor interventions to mitigate the spread of the virus and protect vulnerable populations. It is important to note that the situation is dynamic, and the number of confirmed cases in Indonesia continues to evolve. Therefore, it is essential to refer to the latest data from reputable sources such as the Indonesian Ministry of Health (https://www.kemkes.go.id/) and other international health organizations for the most up-to-date and accurate information.

### Indonesia’s Socio-economy and the Implications on Its Healthcare System

While universal health care in Indonesia has been discussed in Indonesia since its independence in the late 1940s, an actionable insurance concept (Indonesian: Jaminan Kesehatan Nasional or JKN) has only been released in 2014 [23], with implementation and refinements still ongoing. Many political and economic challenges have defined Indonesia’s journey to affordable healthcare [24] and to this day the health care system remains a complex scheme in terms of financing, logistics and technicality. The 6000 inhabited islands of Indonesia are subdivided into 38 provinces with over 500 regencies/cities governing over 80,000 villages and with a partially decentralized fiscal set up, provision of universal, equal health care is a challenge [25, 26].

Notwithstanding, in 2022, 2.5% of the population lived below the international poverty line (with ≤ 2.15US$ per day per capita) and another 20.3% lived in moderate poverty (with ≤ 3.65US$ per day per capita). Significant differences in wealth distribution can be found between urban and rural regions [27], reflecting the disparity in development of local infrastructure and access to health care. Data shows that there is a correlation between lower health outcomes and socio-economic status in rural regions, for example, in mortality rates for children under 5 years [28]. In general, accessing health care in rural regions is connected to significant physical, financial and time barriers, despite prevalence for communicable diseases and risks for accidents being higher in rural provinces as well [29].

Three quarters of the participating primary health care providers are government-mandated community health centers (Puskesmas), while the rest are general practitioners and private hospitals [30]. Of particular note is that there are significant differences in facilities and quality between Puskesmas and private providers. As of 2021, more than 80% of the Indonesian population are covered by JKN. People who do not participate in JKN, must cover their health expenses privately. These private patients are often prioritized in treatment and inpatient beds [30].

A patient referral system (SISRUTE) established in 2016 is theoretically able to guide patients from primary to secondary and subsequently to tertiary care facilities, yet non-adherence to the processes due to lack of commitment and supervision of the healthcare workers have characterized its existence and hampering its full potential [31].

Due to the pandemic's profound economic impact, Indonesia experienced a shift in its income status from upper-middle income to lower-middle income as of July 2021 [32]. In addition, the progress made in poverty reduction was partially reversed, with the poverty rate rising from a record-low of 9.2% in September 2019 to 9.7% as of September 2021 [32].

Indonesia's resurgence to the upper-middle income category of nations has been attributed to a robust post-pandemic recovery, as reported by the World Bank [33]. The country's gross national income per capita has surged to $4580, leading to its reclassification in the upper-middle income rank. This notable improvement marks a significant shift from the previous status of lower-middle income, which Indonesia had maintained for two consecutive years, including 2022 when the gross national income per capita was recorded at $4140 [33]. With this recent economic progress, Indonesia is now back on course to pursue its goal of achieving high-income status.

### Crisis Preparedness Before and During the COVID-19 Pandemic

Tectonically located in a very disaster-prone region, frequent earthquakes, volcanic eruptions, tsunamis and flooding have always demanded crisis preparedness from Indonesia’s people. Aside from natural disasters, the tropical climate facilitates zoonosis and infectious diseases with dengue fever, malaria and rabies being endemic [34], and tuberculosis being the leading infectious cause of death prior to SARS-CoV-2 [35]. The Centre for Health Crises (Pusat Penanggulangan Krisis Kesehatan) was established by the Ministry of Health to respond to disease outbreaks, with preparedness mechanisms conceptualized from district up to national level and institutions like the Red Cross taking over the tasks of preparing communities through health programs and trainings [36]. In 2009 South–East Asia faced an Influenza A (H5N1) pandemic resulting in the maintenance of a National Influenza Pandemic Preparedness Plan in Indonesia, with the WHO supporting with technical assistance [37]. This pandemic influenza contingency plan was used to create a COVID-19 response plan in March 2020 [38]. Despite governmental oversight, interventional measures for epidemic and pandemic preparedness lack overall coordination and resources [36]. In addition, communities fail to prioritize public health issues, because their urgency appears less imminent than in natural disasters.

Even before the onset of the pandemic, Indonesia had only 0.38 physicians per 1000 population, significantly lagging behind Malaysia, Thailand and Vietnam [3]. In an effort to increase the COVID-19 response task force, volunteers were additionally recruited.

Regrettably, a year into the pandemic on May 3rd 2021, 366 deaths among health care professionals had been reported [39] and correlated with the unavailability of protective equipment, such as N95 masks and coveralls [40], linked to the interruption of global supply chains caused by factory shutdowns and export bans [41]. Domestic production and distribution were accelerated to counter dependency on other economies [42].

Other long established structural weaknesses such as non-compliance with the formal patient referral system and infrastructural inadequacies in waste management [43], bed capacities (1.49 hospital beds per 1000 population) and other facilities worsened under the pressure of the COVID-19 pandemic [3].

### Testing

Indonesia has implemented a multifaceted testing strategy to detect and monitor COVID-19 cases. Rapid testing is essential to reduce the basic reproduction number [44]. The testing strategy includes the following key components:

1. Polymerase Chain Reaction (PCR) Testing: PCR testing is the gold standard for COVID-19 diagnosis and is widely used in Indonesia. In the beginning of the pandemic, the only acknowledged method to detect COVID-19 infections rapidly and accurately was via RT-PCR (reverse transcription polymerase chain reaction) [11]. The tests are conducted using respiratory samples, such as nasopharyngeal and oropharyngeal swabs, to detect the presence of the virus’s genetic material [45]. PCR testing provides accurate results and is primarily performed in healthcare facilities, laboratories, and designated testing centers [46].
2. Rapid Antigen Testing: Rapid antigen tests are used as a screening tool to quickly identify individuals who are likely to be infected with COVID-19. These tests detect specific viral proteins and produce results within a shorter timeframe [47]. Rapid antigen tests are often utilized in community-based settings, airports, and other high-risk areas to facilitate faster detection and isolation of potential cases.
3. Targeted Testing: The Indonesian government has implemented targeted testing strategies to identify and test individuals who meet specific criteria. This includes individuals with symptoms consistent with COVID-19, close contacts of confirmed cases, healthcare workers, and high-risk populations, such as elderly individuals and those with underlying health conditions [48, 49]. Targeted testing helps prioritize limited resources and focus efforts on populations at higher risk of infection.
4. Surveillance Testing: Surveillance testing involves conducting tests on a sample of the population to monitor the prevalence of the virus within specific regions or communities. It helps provide a broader understanding of the disease’s spread and identifies potential hotspots. Surveillance testing is crucial for early detection and response to prevent further transmission [50]. For instance, new evidence has shown that increased monitoring through regular testing of personnel at skilled nursing facilities was linked to significant decreases in COVID-19 cases and fatalities among residents, particularly during the period before vaccines became available [51]. Pooled testing combines samples, reducing the number of tests needed. Studies demonstrate it can detect positive samples accurately, making it suitable for population-wide screening and surveillance with existing resources [52]. Surveillance testing with rapid SARS-CoV-2 antigen detection tests is well-received in Indonesia, empowering individuals, including asymptomatic ones, to monitor their COVID-19 status and contribute to community-based surveillance efforts [53]. This approach eases the burden on the healthcare system while enabling timely responses to potential outbreaks. A cross-sectional study in Jakarta has shown that integrating SARS-CoV-2 antibody rapid diagnostic test–IgM/IgG results with other data may offer an affordable approach for epidemiological surveillance, allowing assessment of population-based COVID-19 exposure and current infection, especially in areas with outbreaks or high transmission [54].
5. Scaling Up Testing Capacity: The Indonesian government has taken steps to enhance testing capacity by establishing more laboratories across the country, expanding the network of testing facilities, and increasing the availability of testing kits and supplies [55]. This allows for more widespread and efficient testing to be conducted, leading to improved case detection and management.

Despite being the fourth most populous country of in the world, Indonesia has reported a relatively small number of confirmed COVID-19 cases compared to other affected countries, especially in the early stages of the pandemic [56]. Consequently, many questions were raised concerning the capacity of COVID-19 testing in Indonesia. Low testing capacities led to massive under-reporting, which most likely resulted in the low number of cases [56]. With a case fatality rate (CFR) of 8.9% by the end of March 2020 [57], there is reason to believe that the elevated CFR regarding COVID-19 patients may be attributable to several factors. Primarily, the country’s limited testing capacity, stemming from a severe scarcity of essential molecular testing reagents, could contribute to this outcome. In addition, the lack of adequate healthcare facilities nationwide, coupled with physically and psychologically overburdened healthcare workers who have been tirelessly supporting the national healthcare system [49], might have further exacerbated the situation. Later on, testing was connected with significant fees for the public as it was not offered as a free service [58], decreasing compliance even further.

Given these circumstances, it is highly probable that diagnostic resources were prioritized to examine individuals presenting conspicuous symptoms, rather than ensuring comprehensive coverage of the entire population to detect COVID-19 cases, including those with mild or even asymptomatic manifestations. Therefore, this selective approach could potentially result in the under-reporting of the true number of COVID-19 cases in Indonesia.

By May 19, 2020, Indonesia achieved a significant milestone of conducting 10,000 PCR tests per day. Subsequently, in June 2020, the WHO reported that Jakarta met the minimum criteria of conducting at least one test per 1000 population per week. This requirement ensures a reliable calculation of the positivity rate, which is an important indicator for monitoring the spread of COVID-19 [16].

The government works closely with health authorities to adapt the testing approach based on emerging evidence and international guidelines to ensure effective disease control and prevention measures.

### Vaccination

The Indonesian government’s plan for COVID-19 vaccination was established through the enactment of Presidential Decree number 99/2020 on October 5, 2020 [59]. This decree serves as the legal basis for the procurement and implementation of COVID-19 vaccines. Furthermore, Minister of Health Regulations number 84/2020 on December 14, 2020, provided detailed plans for the implementation, including the selection of vaccine types and vaccination targets. The Government of Indonesia proactively secured vaccines at an early stage, finalizing the procurement in 2020. This early action ensured that Indonesia had an adequate supply of doses to achieve herd immunity. By January 2022, the government had set the target of administering 426 million doses from various sources, including Sinovac Biotech, Novavax, Pfizer, and AstraZeneca, with the aim of vaccinating more than 181 million Indonesian citizens to create herd immunity [59, 60].

The limited availability of vaccines in Indonesia initially led to low coverage during the early stages of the vaccination implementation period up to February 2021. However, with the subsequent increase in vaccine availability, the vaccination rate accelerated significantly from February 2021 [59]. The Indonesian government employed strategic measures, such as utilizing strategic public places, public and private offices, and engaging the private sector, during the second phase of implementation to expedite the vaccination process. On November 21, 2021, Indonesia had achieved a full vaccination rate of 32.2% among its general population [61]. The national vaccination target of Indonesia included the immunization of approximately 234.6 million people and as of August 9, 2023, 203,872,737 people had received the first dose of the vaccine, accounting for 86.87% of the national vaccination target. Out of these 174,946,899 had received a second dose (74.55% of the national vaccination target). 69,243,490 of them had been inoculated with the booster or third dose, while 3,524,526 had received the fourth dose [62].

Healthcare workers in Indonesia are more prone to being exposed to SARS-CoV-2 compared to the general population [63]. In this context, the COVID-19 pandemic presented a multitude of challenges encountered by healthcare professionals within hospital settings [64]. The mortality rate among healthcare workers was shown to be notably greater than that observed in the wider population [65]. It is pertinent to note that the mortality rate among healthcare workers is fivefold higher than that of the general population [65]. In the hopes to alleviate this, the government prioritized health care workers and support staff to be the first to be vaccinated as the vaccines initially entered the Indonesian market [48, 60]. A study has presented precise insights into the incidence, hospitalization rates, and characteristics of COVID-19 cases among healthcare workers in East Java, Indonesia, comparing the periods before and after vaccination [48]. Despite the high vaccination coverage, the study was able to demonstrate that the risk of contracting COVID-19 remains elevated among healthcare workers in two major hospitals in East Java. The occurrence of COVID-19 over a span of 30 days among healthcare workers in those two hospitals rose from 1.57% prior to the initiation of the vaccination campaign to 2.34% subsequent to it. This escalated COVID-19 incidence is potentially linked to the emergence of the Delta variant. Upon meticulous data analysis, it was observed that the occurrence of COVID-19 diminished in the initial 3 months of the vaccination initiative. However, by the end of May 2021, the infection rate experienced a stark escalation, synchronizing with the rise of the Delta variant of the SARS-CoV-2 virus [66]. It is quite plausible that the safeguarding efficacy of the inactivated viral vaccine was significantly compromised against the Delta variant, as evidenced by the notable decline in vaccine effectiveness, plummeting from 73.3% to 17.6% upon the Delta variant's dominance in the province [48]. This observation aligns with earlier findings indicating the diminished vaccine effectiveness against the emerging Delta variant [67, 68].

The public attitudes towards vaccination and the role of vaccine hesitancy in shaping the immunization landscape has also been explored. There is diverging information concerning vaccine acceptance rates around the world. The countries with the highest rates of COVID-19 vaccine acceptance among the adult population are Ecuador (97.0%), Malaysia (94.3%), Indonesia (93.3%), and China (91.3%) [69]. This data contrast with results from another study, with only 78% vaccination acceptance for Malaysia and 67% for Indonesia [70]. The vaccination acceptance in Indonesia (67%) aligns closely with the COVID-19 vaccination status in Indonesia (62.5% completely vaccinated) at the time of the study’s publication [70]. Of note, the acceptance of COVID-19 vaccination, in general, does not necessarily translate into actual vaccination uptake.

The willingness to receive a booster dose in Indonesia was observed to be lower when compared to the acceptance of the initial vaccine prior to its introduction in 2021 [71]. This may be due to several reasons. The lower acceptance of a booster dose in Indonesia could be attributed to pandemic fatigue, as people have become desensitized to continuous news about the pandemic after 2 years of exposure. Repeated unfulfilled promises regarding COVID-19 mitigation strategies may have eroded trust and willingness to follow new preventive behaviors, such as taking a booster dose [72]. A study found that the vaccination service itself divided society almost equally in terms of satisfaction and dissatisfaction [73]. In addition, the relatively lower severity of the Omicron variant among the younger age group [74, 75] has contributed to a false sense of security, reducing the perceived threat of the virus. Moreover, pandemic-related news have experienced a diminished presence in the Indonesian news cycle, overshadowed by other pressing concerns, such as economic, social, and political issues. This situation is exacerbated by the government's comparatively lax response, particularly when juxtaposed with the stringent social restrictions implemented during the Delta wave [76]. This combination of factors may have led to the decline in acceptance and urgency to seek a booster dose among respondents in Indonesia.

However, in hypothetical situations, where boosters were required for work, travel, and accessing public places, acceptance rates increased [71]. To enhance the coverage of booster doses, effective strategies should be implemented, taking into account health beliefs and targeting individuals with lower socioeconomic status. Fostering positive information can transform the perception of community vulnerability, serving as a catalyst for active involvement in the COVID-19 vaccination initiative [77]. This presents an effective strategy to broaden the campaign’s impact and alleviate public hesitations towards vaccine acceptance in Indonesia.

### Mobility, Mass Movement During National Holidays and Large Social Events

Managing mobility and ensuring compliance with health protocols are of utmost importance, particularly during periods of relaxation and public holidays when increased mobility is anticipated. Prolonged pandemic situations and relaxed mobility measures can potentially lead to decreased adherence to both mobility and health protocols. Following the initial relaxation period, there was a gradual rise in mobility trends in Jakarta. However, after the implementation of the second set of restrictions in September 2020, mobility witnessed a decline, only to rise again when relaxation measures were reintroduced in October 2020 [78].

During the pandemic, the Indonesian government introduced Large-Scale Social Restrictions (Indonesian: Pembatasan Sosial Berskala Besar or PSBB), which included measures such as closing public places, restricting public transport, and limiting travel to and from restricted regions [79, 80]. A study has suggested that different types of mobility can be considered as significant predictors for COVID-19 cases and have different levels of impact on COVID-19 dynamics in Jakarta [78].

There is a growing body of evidence indicating that the transmission of SARS-CoV-2 is associated with large social gatherings, e.g., weddings and church services [81–83]. A substantial rate of COVID-19 infection among a group of individuals who attended a wedding event in Bali has been observed [84]. These attendees were involved in close physical interactions, shared beverages, and maintained close proximity to one another during the festivities. These factors likely contributed to the elevated rate of infection within this cohort. This outbreak serves as a notable example of how social events can play a significant role in the spread of COVID-19. It emphasizes the importance of restricting gatherings and minimizing close physical contact as crucial measures in controlling the transmission of the virus.

During the Lunar New Year and Eid al-Fitr, both of which are celebrated as national holidays in Indonesia, there is a significant influx of people traveling and gathering. Mudik, an Indonesian tradition of annual homecoming during the celebration of Eid Al-Fitr, involves the migration of millions of individuals from one region to another. This event typically occurs after the fasting month of Ramadan. Similar to China’s Lunar New Year migration, Mudik is recognized as a high-risk activity [85, 86]. In an effort to curb the spread of SARS-CoV-2, the Indonesian government prohibited this traditional practice in both 2020 and 2021 [87]. However, in 2022, the ban was lifted, resulting in approximately 6.3 million individuals utilizing public transportation for Mudik and traveling across Indonesia [88]. Most of the traffic was observed from Jakarta to various cities on the island of Java. Following the Mudik period, a counter-migration event, known as Contra-Mudik, took place on May 10, 2022, as people returned to Jakarta. During this time, the Indonesian government waived the requirement for PCR testing when using public transportation or engaging in international travel. Instead, the focus was shifted towards ramping up vaccination efforts, with more than 60% of the Indonesian population receiving their vaccinations. The Lunar New Year celebration in 2020 played a role in facilitating the transmission of COVID-19, while the Eid al-Fitr celebration in 2022 served as a crucial assessment of the federal government’s management of the pandemic and the effectiveness of the vaccination campaign in Indonesia [88].

On the whole, it is crucial to adapt or even impose restrictions on activities involving close interaction, such as traditional markets, religious gatherings, and wedding parties, as these settings can become significant sources of virus transmission. By taking proactive measures in such settings, the risk of COVID-19 transmission can be mitigated effectively.

### The Impact of COVID-19 on Work Life in Indonesia

The COVID-19 pandemic has changed people’s social–economic life pillars. COVID-19 brought significant impacts on occupations in the form of delayed, declined and even lost work. Indonesians who carried out self-isolation possibly faced big problems, since they then lived at home, ceased working, and had no income (many do not have permanent income) and could not directly interact with their social environment [89]. In this context, the psychosocial aspects and psychiatric sequelae of COVID-19 for the Indonesian community are also essential [90, 91].

The concept of remote work/working from home has had a notable impact on corporate culture and productivity in Indonesia [92]. The ongoing coronavirus pandemic has provided valuable insights to some employers regarding its implications for their businesses and employees, even if remote work is not currently feasible for their workforce. Embracing remote work offers several advantages for organizations, including increased flexibility, agility, improved employee retention, access to new talent, heightened productivity, and enhanced staff motivation [93]. However, there are certain challenges associated with remote work for employees. Not everyone may be suited to this style of work, and individuals may experience feelings of isolation. Monitoring performance can be more difficult, distractions at home can hinder productivity, and there is a risk of burnout and negative impacts on mental health [94]. While the benefits of remote work are significant, it is important to address and find solutions for the potential drawbacks.

In particular, remote working is only possible for limited lines of work and in Indonesia, that is the minority. With more than 60% of the Indonesian workforce securing their livelihood in the informal sector [95], the impacts of remote work need to be relativized as government-implemented decrees on working from home meant partial or complete loss of income for this group as many informal jobs can be found in trade, tourism, gastronomy, public transportation, domestic work and other services—all industries that required social interaction [96]. Prior to the COVID-19 pandemic, this heterogenous group of workers was already subject to vulnerable employment and unstable income, yet were not considered to be “poor enough” to qualify for government help programs during the pandemic and those who did qualify for help programs were difficult to identify, since informal workers are unregistered and high migration and mobility among this group is not documented. The informal sector has been significantly affected by the COVID-19 pandemic, resulting in notable consequences, such as reduced working hours, a decline in the number of informal sector workers, and a decrease in income [97]. The repercussions of the pandemic began to manifest shortly after the emergence of COVID-19 in Indonesia and the subsequent implementation of extensive social restrictions by the government. Confronted with this unprecedented crisis, workers in the informal sector have predominantly adopted a livelihood strategy characterized by sacrifice, particularly through the implementation of cost-saving measures within their households. Unfortunately, the adverse impact of the pandemic on their livelihoods has led many of them to rely on social assistance, which, regrettably, remains inaccessible to a significant portion of this population [97]. Finally, as data on the informal sector is scarce, the exact impact of the COVID-19 pandemic on this group remains largely unknown [98].

### COVID-19 Complications, Therapy, and Potential Complementary and Alternative Medicine (CAM)

In addition to the widely known respiratory symptoms [99], SARS-CoV-2 has the potential to cause various other complications, e.g., cardiovascular [100], neurological [101], (neuro)psychiatric [102], dermatologic [103] etc. Although extensive global endeavors have been undertaken to effectively address the COVID-19 crisis, the search for a definitive and targeted treatment for COVID-19 remains ongoing. The standard treatments for managing the disease involve antiviral agents [104], anti-inflammatory medications, and respiratory support [105]. Numerous clinical trials are currently investigating diverse therapeutic approaches encompassing a range of medications, convalescent plasma therapy, monoclonal antibodies, immunoglobulin therapy, and cell-based therapies [106].

In the early stages of the worldwide COVID-19 pandemic, there were indications of certain individuals experiencing enduring symptoms and encountering new health issues long after the initial phase of infection, without any apparent explanation from other factors [107]. Recognizing and reporting this novel condition, the patient community coined the term “long COVID” to describe the lingering and prolonged consequences of SARS-CoV-2 infection [108, 109]. Individuals experiencing “long COVID” suffer from a diverse array of physical and mental symptoms, while they often express challenges related to their quality of life, mental well-being, and employment. Given these circumstances, comprehensive care involving continuous symptom monitoring, identification of potential complications, physical rehabilitation, mental health support, and social services may be necessary. Therefore, alongside the prevailing approaches centered on preventing COVID-19 infection and addressing acute symptoms, there is a burgeoning interest in the long-term management of symptoms. In resource-constrained countries, such as Indonesia, there exists the potential for the underutilization of internationally recognized treatment strategies due to inadequate healthcare resources. Consequently, complementary and alternative medicine (CAM) interventions are being explored as alternative modalities for COVID-19 management, taking into consideration the specific medical context of each country [110–112]. For instance, phytochemicals hold significant potential as novel chemical leads for the creation of safe and potent anti-SARS-CoV-2 agents [113]. Microbial natural products with possible antiviral activities have also been evaluated [114]. Indonesia possesses a rich abundance of medicinal plants, deeply rooted in its historical tradition of utilizing plant-based remedies for various hereditary diseases. In line with this, the Indonesian government actively encourages the exploration of domestic resources for addressing the challenges posed by COVID-19. The primary focus of available potential products is aimed at bolstering and enhancing the human immune system. The prospects of natural compounds sourced from Indonesian medicinal plants in the context of COVID-19 encompass various examples, including trans-cinnamaldehyde and related analogs derived from *Cinnamomum verum*, which exhibit immunomodulatory properties [115]. In addition, substances like curcumin and polysaccharides act as immunomodulators, alongside phyllanthin and related phenolic compounds sourced from *Phyllanthus niruri* [116]. Moreover, polysaccharides derived from *Oryza sativa* also exhibit immunomodulatory effects [117].

In general, there is a need for further enhancement in the level of evidence regarding the effectiveness of CAM in treating COVID-19. Several case studies have already been conducted to assess the effectiveness of phytochemicals as anti-SARS-CoV-2 agents in human subjects [113]. A systematic review and meta-analysis have shown that in the realm of COVID-19 management, herbal medicine presents itself as a promising adjuvant therapy with substantial potential [118]. Furthermore, available evidence suggests that Traditional Chinese Medicine medications demonstrate efficacy in alleviating symptoms among patients with COVID-19 [110]. However, to establish the true efficacy of CAM in treating COVID-19, more systematic and controlled clinical studies are warranted.

The abundant biodiversity found in Indonesian flora offers a diverse array of structural compounds derived from natural sources, which serve as valuable assets in the pursuit of identifying potential drug candidates for combating SARS-CoV-2 infection [119].

## Conclusions

The COVID-19 pandemic has presented unique challenges for Indonesia, an archipelago consisting of thousands of islands. Indonesia’s geographical complexity adds a layer of intricacy to its pandemic response efforts. With over 17,000 islands (out of which around 6000 are inhabited) spanning vast distances and diverse demographics and cultural practices, there are many factors that contribute to the complicated landscape of the pandemic, requiring tailored strategies and nuanced approaches.

The archipelagic nature of Indonesia has also posed challenges in testing and contact tracing. The limited laboratory capacities in certain regions have resulted in variations in testing capabilities and delays in obtaining test results. Moreover, the vast distances between islands have made it difficult to swiftly identify and trace contacts of infected individuals, leading to potential gaps in the surveillance system.

Moving forward, it is crucial for Indonesia to focus on increasing public awareness and adherence to health protocols. Tackling this issue would require profound educational effort, as education as an element of health is closely linked to developing cognitive functions that can subsequently conceive public health as a priority [120]. Training healthcare professionals to adhere to formal processes may improve effectiveness of already established systems. To facilitate health equity in Indonesia promoting equal healthcare and vaccine distribution across the archipelago should be striven for. As a first measure to reach more remote regions, a telemedicine system may provide a transitional remedial action.

The socio-cultural aspects of Indonesia have also played a role in the response to the pandemic. Traditional ceremonies, religious gatherings, and close-knit community interactions are deeply ingrained in Indonesian culture, making it difficult to enforce physical distancing measures and limit social gatherings. Cultural practices, such as *Mudik* (annual homecoming), have posed significant risks for the spread of the virus during festive seasons. Here too, comprehension of the necessity to put traditions aside for public safety, may eventually be achieved through better quality education.

The government had taken various steps to address these problems, including the implementation of public health regulations, travel restrictions, and vaccination campaigns, yet remediating basic structural inadequacies may need to take precedence, now that the urgency of a global health crisis has diminished and left room to improve and learn from past experiences.

This review article aims to contribute to the ongoing discourse surrounding the COVID-19 situation in Indonesia, offering valuable perspectives on the obstacles encountered and the strategies employed. By highlighting the unique aspects of Indonesia’s pandemic experience, we hope to foster a deeper understanding of the complex dynamics at play and inspire dialogue on how to navigate the remaining uncertainties and chart a path towards resilience and recovery.

## Data Availability

Authors can confirm that all relevant data are included in the article. Data set(s) derived from public resources and made available with the article (references).

## Abbreviations

COVID-19: Coronavirus disease 2019
SARS-CoV-2: Severe acute respiratory syndrome coronavirus 2
WHO: World Health Organization
PHEIC: Public Health Emergency of International Concern
JKN: Jaminan Kesehatan Nasional
SISRUTE: Sistem Informasi Rujukan Terintegrasi Nasional
PCR: Polymerase chain reaction
RT-PCR: Reverse transcription polymerase chain reaction
CFR: Case fatality rate
CAM: Complementary and alternative medicine
